# Retained Bullet in the Penis

**DOI:** 10.7759/cureus.16488

**Published:** 2021-07-19

**Authors:** Morgan Eudy, Natalie Nowak

**Affiliations:** 1 Surgery, Campbell University School of Osteopathic Medicine, Salisbury, USA; 2 Trauma and Acute Care Surgery, Team Health, Salisbury, USA

**Keywords:** gun-shot wound, penile trauma, retained bullet, penile injury, genitourinary trauma

## Abstract

Gunshot injuries to the genitourinary system are extremely rare among trauma cases and represent a complex clinical scenario to physicians. This rare case involves a 20-year-old male with a retained bullet in his penis following a close-range, low-velocity gunshot wound to the right lateral thigh. Our case report examines this unique clinical scenario and the diagnostic approach that should be taken to decrease the chances of negative cosmetic and functional outcomes.

## Introduction

Genitourinary injuries are rare among trauma cases and only constitute <10% of all cases, with the penis being involved in 10-16% of those [1]. Complications from gunshot wounds to the penis can include bleeding, infection, penile curvature, erectile dysfunction, and urethral stenosis [2]. Evaluation for a gunshot wound to the penis should begin by ruling out injury to the corpora cavernosa and urethra [3].

Signs of injury to the urethra include hematuria or the presence of blood at the urethral meatus, but these are not specific findings and it is recommended that a retrograde urethrogram should be performed on all patients to rule out urethral injury [1,3]. Reports of urethral injury vary widely, with one study showing urethral injury constituting 23% of gunshot wounds to the penis [3]. 

Signs of injury to the corpora cavernosa include a wound on the penile shaft with a palpable corporeal defect, uncontrollable bleeding, or expanding hematoma [3]. Should the corpora cavernosa be injured, time is of the essence to avoid long-term complications [4]. In studies looking at penile fractures, Bozzini et al. found that surgical intervention in less than eight hours was ideal to minimize long-term complications, including penile curvature and erectile dysfunction [4].

## Case presentation

Our patient is a 20-year-old male who was brought in by emergency medical services (EMS) to the Emergency Department with a gunshot wound to the right thigh and a tourniquet in place. 

**Figure 1:**
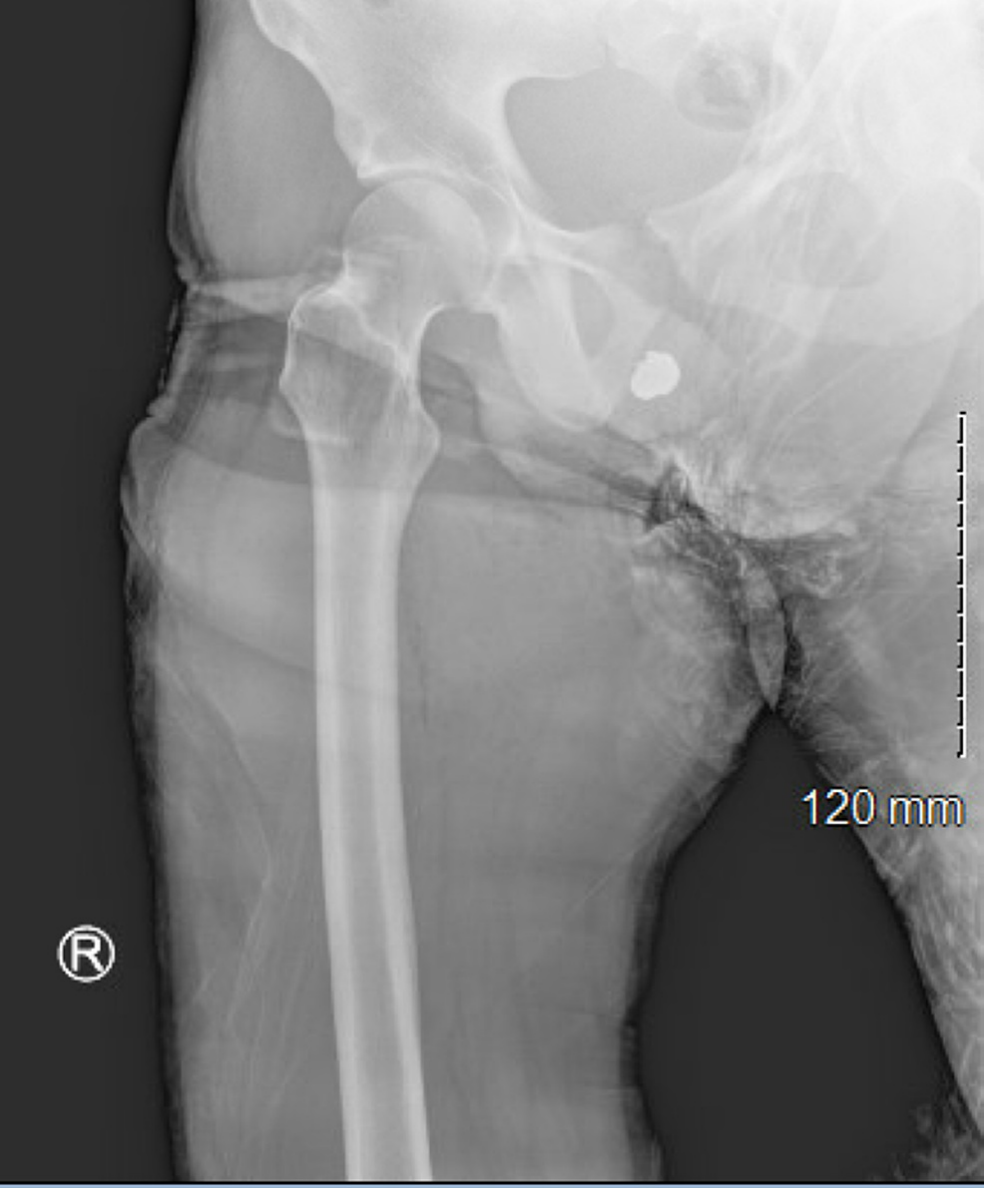
Anterior/Posterior view X-Ray showing retained bullet overlying the groin.

At the time of initial evaluation, the patient was hemodynamically stable. He reported pain in his right leg and penis. Focused physical exam findings revealed lateral and medial wounds on the right proximal thigh that were hemostatic, in addition to a wound to the right lateral ventral penis. The patient was neurovascularly intact distally and there was no concern for compartment syndrome. A lower extremity X-ray reported no evidence of a fracture but did show a retained foreign body (Figure 1). There was tenderness with manipulation of the penis, but no foreign body could be visualized or palpated due to swelling.

**Figure 2:**
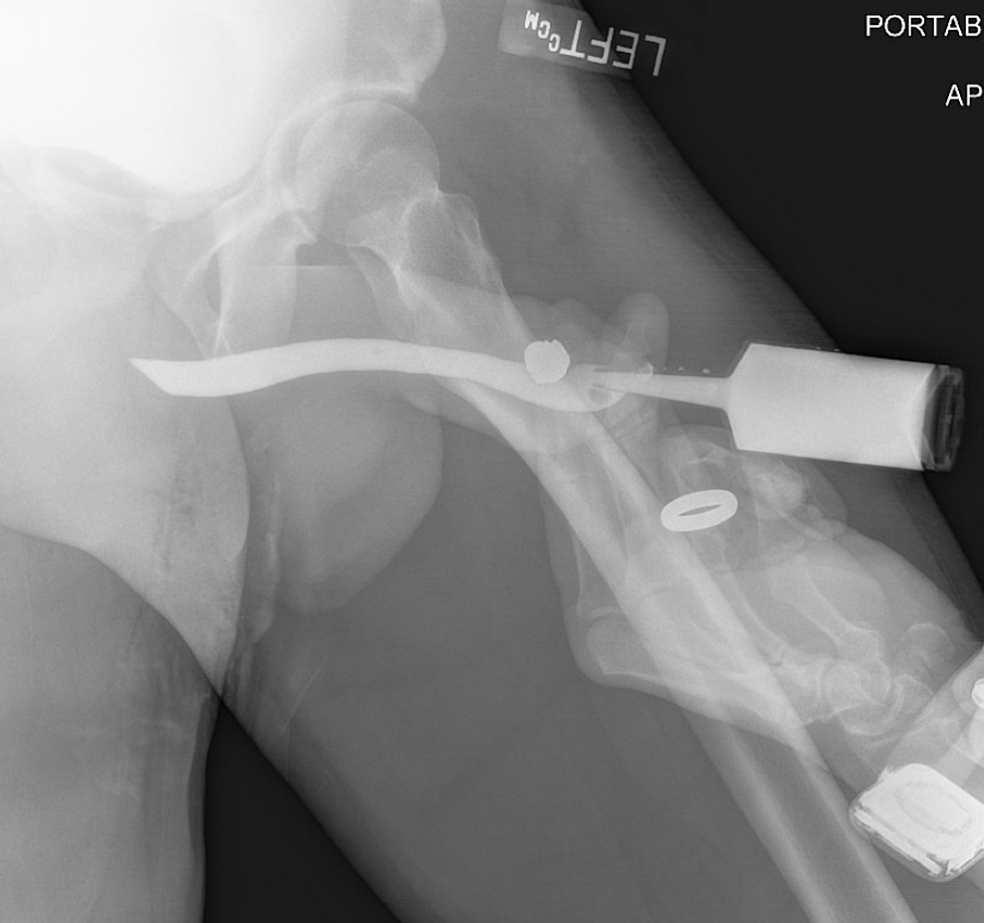
Retrograde urethrogram demonstrating an intact urethra evident by no extravasation of contrast. Also seen is the location of the retained bullet in the shaft of the patient's penis.

After a full exam was performed and no additional injuries were noted, the tourniquet was removed and popliteal and dorsalis pedis pulses were palpated and intact. The patient then underwent a CTA which revealed an occluded major branch of the right profunda femoris artery without active extravasation or visible hematoma. A consultation was made to vascular surgery and it was determined no vascular intervention was needed and aspirin 81 mg daily was recommended. The CTA did show that the bullet was lodged in the head of the penis. A retrograde urethrogram was performed to evaluate for urethral injury. Upon completion, there was no evidence of contrast extravasation (Figure 2). The patient was subsequently taken to the operating room by urology for removal of the bullet, debridement, and primary repair of the right corporal injury. The bullet tracked subcutaneously and did not injure the glans penis or urethra. 

Post-operatively, the patient remained stable and the wounds were hemostatic. The foley catheter was removed eight hours post-operation and the patient was able to urinate with minimal post-void residual volume. There was residual hematuria and the patient was appropriate for discharge. At a two-week follow-up with urology, the patient reported urinating well and achieving erections.

## Discussion

This case is unique due to the bullet being retained in the head of the penis. Typically, gunshot wounds to the penis are through injuries, so having a retained bullet within the penis is a unique clinical scenario. During our review of the literature, we were able to find three other case reports similar to our patient in which the bullet was actually retained within the penis [5-7]. One of the cases involved an entry wound to the left buttocks with the bullet tracking to the penis but not exiting [5]. Another case involved the bullet passing through another individual's abdomen and then lodging within the penis of the man standing behind him [6]. In Saudi Arabia there was a case similar to our patient in which the bullet initially entered and exited the upper thigh before finally lodging within the penis [7].

Overall, gunshot injuries to the penis are extremely rare and are typically seen in battlefield trauma or in large urban areas with criminal implications. The nature of these injuries in these two populations are very different due to military wounds being high velocity, resulting in much more devastating tissue injury. Civilian gunshot wounds are typically low velocity, occurring at less than 1000 feet per second [1]. 

In our research, we discovered that long-term follow-up on penile gunshot wounds is minimal in civilian injuries. Wolfgang et al. equated this to the criminal implication of gunshot injuries and the difficulty in obtaining follow-up in this population, leaving us with a lack of data on erectile dysfunction and cosmetic results [1]. Patients not experiencing erectile dysfunction or cosmetic issues are also commonly lost to follow-up since they are not experiencing issues, generating a bias in prognosis data. Kunkle et al. reported excellent potency and cosmetic results in their study of 63 patients despite a general lack of long-term follow-up data [3]. It is generally agreed upon that quick intervention with reconstruction of the corpora cavernosa is vital in minimizing long-term complications including erectile dysfunction and penile curvature [3-4].

## Conclusions

While gunshot wounds to the penis are extremely rare, having a methodical approach to treating these types of injuries is vitally important. When evaluating these injuries, the clinician should maintain a low threshold of suspicion for cavernosa and urethral injuries. Retrograde urethrogram should be done on all patients to rule out damage to the urethra, except for the most superficial of injuries. Primary closure of the corpora cavernosa in a timely manner is vitally important to decrease the chances of negative cosmetic and functional outcomes. 
